# Changes in Oleic Acid Content of Transgenic Soybeans by Antisense RNA Mediated Posttranscriptional Gene Silencing

**DOI:** 10.1155/2014/921950

**Published:** 2014-08-13

**Authors:** Ling Zhang, Xiang-dong Yang, Yuan-yu Zhang, Jing Yang, Guang-xun Qi, Dong-quan Guo, Guo-jie Xing, Yao Yao, Wen-jing Xu, Hai-yun Li, Qi-yun Li, Ying-shan Dong

**Affiliations:** Agro-Biotechnology Research Institute, Jilin Academy of Agricultural Sciences, Changchun, Jilin 130033, China

## Abstract

The Delta-12 oleate desaturase gene (*FAD2*-1), which converts oleic acid into linoleic acid, is the key enzyme determining the fatty acid composition of seed oil. In this study, we inhibited the expression of endogenous Delta-12 oleate desaturase *GmFad2-1b* gene by using antisense RNA in soybean Williams 82. By employing the soybean cotyledonary-node method, a part of the cDNA of soybean *GmFad2-1b* 801 bp was cloned for the construction of a pCAMBIA3300 vector under the soybean seed promoter *BCSP*. Leaf painting, LibertyLink strip, PCR, Southern blot, qRT-PCR, and fatty acid analysis were used to detect the insertion and expression of *GmFad2-1b* in the transgenic soybean lines. The results indicate that the metabolically engineered plants exhibited a significant increase in oleic acid (up to 51.71%) and a reduction in palmitic acid (to <3%) in their seed oil content. No structural differences were observed between the fatty acids of the transgenic and the nontransgenic oil extracts.

## 1. Introduction

Vegetable oils form an important part of the human diet, providing concentrated sources of energy and essential nutrients. A process is used for the deacidification of a vegetable oil in which the major acid of the vegetable oil is from the group comprised of epoxy fatty acids, hydroxy fatty acids, linoleic acid, and oleic acid [1]. The consumption of oils with high oleic acid content is beneficial because this monounsaturated fatty acid not only improves the shelf life but also reduces the need for hydrogenation, a process adding to the cost of the oil as well as generating unwanted trans fat that has been linked to many health problems in humans [2–4]. The commodity soybean oil is composed of five fatty acids: oleic acid (18:1), palmitic acid (16:0), stearic acid (18:0), linoleic acid (18:2), and linolenic acid (18:3). The percentages of these five fatty acids in soybean oil average are 18%, 10%, 4%, 55%, and 13%, respectively [5]. Most polyunsaturated fatty acids, up to 90% in nonphotosynthetic tissues of plants, are synthesized through a (18:1) desaturase in the endoplasmic reticulum. The endoplasmic reticulum-associated oleate desaturase* FAD2* (1-acyl-2-oleoyl-sn-glycero-3-phosphocholine Δ 12-desaturase) is the key enzyme responsible for the production of linoleic acid in plants [6–8].

In soybeans, two copies of microsomal *ω*-6 desaturase,* FAD2*-1 and* FAD2*-2, have been cloned [9]. Studies have shown that* FAD2*-1 was expressed primarily in the development of seeds, while* FAD2*-2 was expressed in both the vegetative tissues and the development of seeds [10, 11]. Also, the* FAD2*-2 desaturases consisted of* FAD2*-2A (Glyma19g32930),* FAD2*-2B (Glyma19g32940), and* FAD2*-2C (Glyma03g30070).* FAD2*-1A and* FAD2*-1B are most closely related to one another, with a shared genomic organization containing a single intron and a 99% identity in encoded amino acid sequence. Thereby, this confirmed the importance expression during peak oil synthesis, while a possible role was also revealed for* FAD2*-2C under cool temperature conditions [12–14]. Genetic modification (GM) aimed at regulating the* FAD2* expression has been applied to produce oils with a higher C18:1 in soybeans [15–19]. For example, by antisense suppression of* FAD2* in soybeans, a transgenic line was obtained that produced oil with a higher C18:1 (57%) compared to the wild variety [20]. Similarly, downregulating* FAD2*-1 and* FatB* using interference RNA has enabled the production of soybeans with a significantly higher oleic acid content (up to 94.58%) and reduced levels of palmitic acid (<3%) [21]. Recently, Plenish high oleic soybeans have been in commercial production by DuPont since 2012. They were developed using a biotech process known as gene silencing [22]. These results demonstrate that the* GmFAD2 *family members play a very important role in metabolically engineered oil seed plants [23].

In this study, we report the transformation of soybean with* GmFad2-1b* gene by antisense RNA. We analyzed and investigated the fatty acid composition of the transgenic lines and also discussed the nature of the transcript produced by* GmFad2-1b*.

## 2. Materials and Methods

### 2.1. Plant Materials and Cotyledonary-Node Method

Soybean cultivars [Glycine max (L.) Merrill cv. “Williams 82”] were grown in a greenhouse at the Jilin Academy of Agricultural Sciences, Gongzhuling, during a 16 h photoperiod at a 35°C daytime temperature and a 25°C night-time temperature. The cotyledonary-node method used in this study followed the procedure described by Paz et al. [24]. In brief, soybean seeds were surface-sterilized by placing seeds into a tightly sealed chamber containing chlorine gas for 16 h. The sterilized seeds were soaked with sterile water at 28°C during an 18 h photoperiod for 8–12 h. The imbibed soybean seeds were placed on sterile filter paper and cut longitudinally along the hilum. The cotyledons and hypocotyls (~5 mm) from a single seed were split evenly into two explants. Approximately 50 coat-removed explants were then inoculated in a liquid cocultivation medium (CCM), which contains 1/10 B5 salts and vitamins, 3.9 g/L 2-[N-morpholino] ethanesulfonic acid (MES), 3% sucrose (30 g/L) (pH 4), filter-sterilized 0.25 mg/L gibberellic acid (GA3), 400 mg/L L-cysteine, 1.67 mg/L N-6-benzylaminopurine (BAP), 154.2 mg/L dithiothreitol (DTT), and 200 *μ*mol/L acetosyringone (As) for 30 min. After this inoculation, seven explants (adaxial side up) were randomly placed in Petri dishes (90 mm in diameter × 15 mm deep) on sterile filter paper, placed on solid CCM containing 0.5% purified agar (BD-Difco), and incubated in the dark at 25°C for 3–5 days.

After the cocultivation, the explants were then transferred into solidified shoot induction (SI) medium containing B5 salts and vitamins, 3% sucrose, 0.8% agar (8 g/L; Sigma), 0.58 mg/L MES (pH 5.8), filter-sterilized 1.67 mg/L BAP, 250 mg/L ticarcillin (Tic), 100 mg/L cefotaxime (Cef), and 5 mg/L glufosinate and then incubated in a growth room at 28°C under an 18 h photoperiod for two weeks. Then, the hypocotyl and shoots were trimmed from the explants and the remaining cotyledons with developing nodules were subcultured to a fresh SI medium for another two weeks. Four weeks after SI, the cotyledons were removed from the explants and the remaining tissues were transferred into a shoot elongation (SE) medium. The SE medium was composed of MS salts and vitamins, 3% sucrose, 0.8% agar (Sigma), 0.58 mg/L MES, filter-sterilized 50 mg/L L-asparagine, 50 mg/L glutamine, 0.1 mg/L indole acetic acid (IAA), 0.5 mg/L GA3, 1 mg/L zeatin riboside (ZR), 250 mg/L Tic, 100 mg/L Cef, and 5 mg/L glufosinate (pH 5.8). The explants were then transferred to fresh SE medium every two weeks until the regenerated shoots were suitable for rooting. The incubation conditions were the same as the conditions for SI. At this point, elongated shoots (3-4 cm in length) were excised and placed into a rooting medium (RM) containing MS salts and vitamins, 3% sucrose, 0.8% agar (Sigma), 0.58 mg/L MES (pH 5.8), filter-sterilized 50 mg/L L-asparagine, 50 mg/L glutamine, 0.1 mg/L indole butyric acid (IBA), 250 mg/L Tic, and 100 mg/L Cef. After a period of 1-2 weeks, the roots were fully developed to 2-3 cm in length and eventually transplanted into the pots to be grown in the greenhouse. The transgene events were named by EB8001 to EB8067. The T0 generation transgenic plants were grown in the greenhouse and the T_1_ and T_2_ lines were grown under field conditions.

### 2.2. Nucleic Acid Isolation and Vector Construction

The fresh soybean tissues were collected to extract the total RNA with TRIZOL reagent (Invitrogen). RNA samples (2 *μ*g) were treated with the amplification grade DNase I (Invitrogen) at 37°C for 15 min prior to cDNA synthesis using SuperScript III Reverse Transcriptase (Invitrogen) according to the manufacturer's instructions.

For the assembly of the* GmFad2-1b* antisense RNA vector, a part of a fragment about 801 bp of the* GmFad2-1b* (GenBank accession: XM_003555831) was amplified from the cDNA above by using the primers GmFad2-1b-F/GmFad2-1b-R (Table 1). The amplified* GmFad2-1b* gene product was cloned into the pEASY-T1 cloning vector (TransGene). The product was sequenced using T7 and M13 vector sequencing primers to confirm the sequence of the ligated product. An 801 bp* Xba*I/*Sac*I T1-vector digested* GmFad2-1b* fragment was anticloned into a pCAMBIA3300 vector and digested with the same enzymes to put the* GmFad2-1b* gene under the seed *α*′ subunit of the *β*-conglycinin promoter (*BCSP*) with the different direction, and the *BCSP*′ function was discussed by Imoto et al. [25]. The pCAMBIA3300 binary vector has a kanamycin resistance gene for bacterial selection and a phosphinothricin acetyl transferase (*bar*) resistance gene for plant selection. The* bar* gene was driven by the constitutive CaMV 35S promoter. The* Agrobacterium tumefaciens* strain EHA101 was used in this study.

### 2.3. Detection of Transgenic Soybean Plants

Transgenic soybean plants were verified by leaf painting, LibertyLink strip analysis, polymerase chain reaction (PCR) analysis, and Southern blot. Leaf painting was performed following the procedure described by Ma et al. [26]. Stock of LibertyLink Basta (135 g/L) was diluted by 1000-fold and painted on half the upper surface of the tested soybean leaves. After 3–5 days, the painted leaves of the negative plants died and positive plants remained healthy. LibertyLink strips (Envirologix) were used to determine the genetically modified plants containing the phosphinothricin N-acetyltransferase (PAT) protein following the manufacturer's instructions. To summarize, a circular leaf tissue was isolated by closing the cap of the Eppendorf tubes. The tissue was ground with a pestle for 20–30 sec and 0.25 mL of protein extraction buffer was added before regrinding. The development of the control line indicated that the strip had functioned properly. The second line (test line) would show up when the tested sample was positive.

For the PCR analysis, the genomic DNA of transgenic soybeans was extracted using a simple and quick DNA extracting method by Edwards et al. [27]. We designed the detection primers named EB-R/F according to the* GmFad2-1b* gene and the upstream* BCSP* promoter sequence. The length of the product was 752 bp. The primer sequence can be seen in Table 1. The PCR reaction was conducted using an initial denaturation at 94°C for 5 min, followed by 30 cycles of 94°C for 45 sec, 58°C for 45 sec, and 72°C for 1 min and a final extension of 10 min at 72°C. The PCR products were analyzed on 1.0% agarose gels. As a positive control, a* GmFad2-1b* gene expression cassette containing pCAMBIA3300 vector DNA was also used. The genomic DNA from the empty pCAMBIA3300 vector transformed plant and the untransformed Williams 82 were used as negative controls.

A Southern blot analysis of the PCR positive plants was performed by DIG High Prime DNA Labeling and Detection Starter Kit II (Roche, cat. number 11585614910) according to the manufacturer's instructions. The genomic DNA of transgenic T_1_ and T_2_ plants was isolated by using the high-salt CTAB DNA method. In summation, 20 *μ*g of genomic DNA was digested with* Xba*I and* Hind*III electrophoresed on 1.0% agarose gel, respectively. The DNA was denatured by an alkaline solution and transferred to positively charged Amersham nylon membranes (Amersham) according to standard protocol and fixed by UV-crosslinking at 1200 U for 1 min. Since the* GmFad2-1b* gene and seed-specific promoter* BCSP* were endogenous genes, we took the marker gene* bar* in pCAMBIA3300 vector as the probe for the Southern blot. The probe was labeled using digoxigenin- (DIG-) 11-dUTP with DIG High Prime DNA Labeling Reagents (Roche). Hybridization was carried out at 42°C. Washing, blocking, and detection were carried out according to the manufacturer's instructions. The Southern blots were then exposed at room temperature for 15–20 min and subsequently developed.

### 2.4. Fatty Acid Analysis

The fatty acid compositions of the seeds harvested from T_1_ and T_2_ transgenic plants were analyzed by gas chromatography (GC). First, the lyophilized soybean seeds were ground to a fine powder, and then the lipids were extracted with heptane. The supernatant was then transferred into a glass vial, and the heptane was evaporated with a flow of dry nitrogen gas at 80°C. At this point, an 8 mg aliquot of the extracted soybean oil was transesterified with sodium methoxide. The fatty acid methyl esters were then separated on a Hewlett-Packard 6890 GC supplied with a hydrogen flame ionization detector and a capillary column HP INNOWAX (30 m; 0.25 mm i.d.) with an N2 carrier at 2 mL/min. The oven temperature was maintained at 100°C for a minimum of 1 min and then increased linearly to 250°C (15°C/min). The fatty acid methyl esters (FAMEs) from TAG were identified by comparison of their retention times with the known standards (37-component FAME mix, Supelco 47885-U). Relative fatty acid compositions were then calculated as the percentage that each fatty acid represented of the total measured fatty acids.

### 2.5. Quantitative RT-PCR Analysis

The total RNA was extracted by using a TRIZOL reagent kit (Invitrogen) from the different issues of the mature T_2_ transgenic plants and wild types. At this point, two *µ*g total RNA from each sample was reverse-transcribed by using the PrimeScript RT reagent kit (Takara) with an oligo (dT) 18 primer. Quantitative PCR was performed on the real-time PCR system (Applied Biosystems, 7900) with SYBR Premix Ex Taq (Takara), with the conditions of 95°C for 30 sec, followed by 40 cycles of 95°C for 5 sec and 60°C for 30 sec. The GmActin gene (NM_001289231) was used as a positive internal control and the gene primers for the qRT-PCR were designed using conserved sequences for them as listed in Table 1.

## 3. Results and Discussion

**Figure 1:**
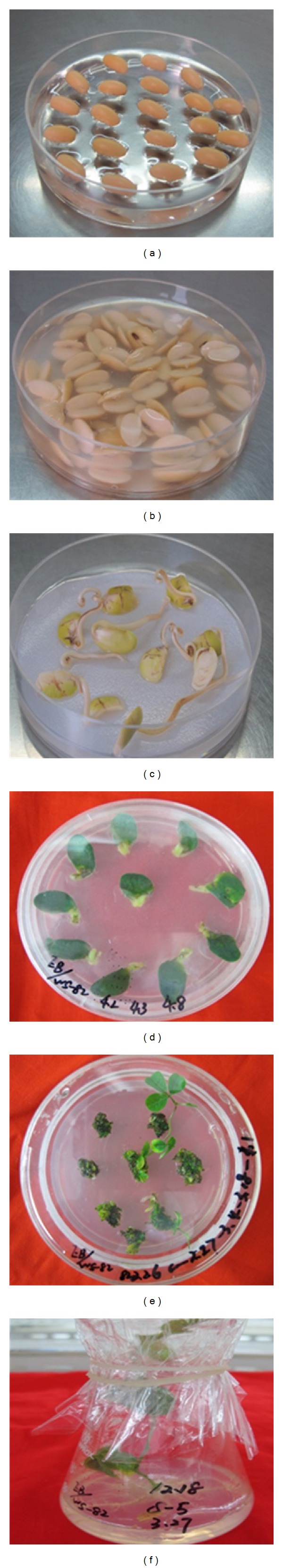
*Agrobacterium*-mediated soybean transformation using the cotyledonary node as explants. (a) Seed germination in the GM medium for the night. (b) Inoculation of explants with* Agrobacterium*. (c) A longitudinal cut between the cotyledons and through the hypocotyl to generate two identical explants. (c) Explants cocultured with* Agrobacterium *for 5 days. (d) Two weeks after selection on shoot induction (SI) medium containing 5 mg/L glufosinate. (e) Elongated shoot in shoot elongation (SE) medium. (f) Rooting of the resistant shoot.

**Figure 2:**
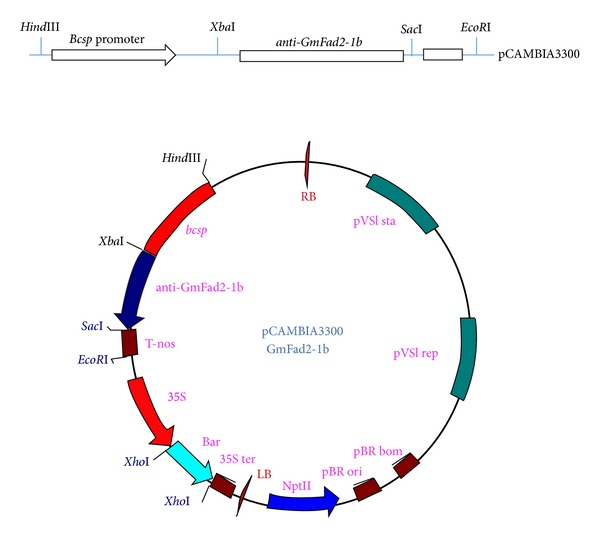
Map of vector pCmbia3300-*Bcsp-GmFad2*. A schematic representation of the expression cassettes of the anti-GmFad2-1b and pCAMBIA3300 plasmids used for* Agrobacterium*-mediated transformation of soybean cotyledonary node. The anti-GmFad2-1b gene is under the control of the soybean seed promoter and 30 region (terminator).

The high-frequency plant gene transfer system is one of the key stages for introducing useful or novel gene(s) into soybeans. In this study, we used the cotyledonary explant derived from mature soybean seeds for* Agrobacterium*-mediated transformation. The entire procedure for the* Agrobacterium*-mediated cotyledonary-node transformation system used in this study is illustrated in Figure 1 and Table 2. Additionally, high-level expression of recombinant molecules has been consistently achieved by utilizing specific regulatory sequences within the seed tissues. The cobombardment transformation strategy to generate transgenic soybean plants allowed us to evaluate the pCAMBIA3300-*BCSP*-anti-*GmFad2-1b* plasmid vector (Figure 2). The gene encoding of the anti-*GmFad2-1b* was placed under the control of *α*′ subunit of the *β*-conglycinin (*BCSP*) soybean promoter and was introduced into the soybean genome with the aim of high-level suppressions, which resulted in a high oleic acid content in the soybean seeds. The promoter* BCSP* is tissue-specific and temporally regulated and is not expressed in the roots, stems, or leaves of soybean. In this study, the gene* GmFad2-1b* under promoter* BCSP* was not expressed in the seed of soybean as expected from the result of the qRT-PCR assay (Figure 5). Regulatory information for the tissue-specific expression mediated by the* BCSP* promoter and the other elements involved in the modulation of the expression of genes associated with seed storage protein has been localized to the DNA sequences approximately 250 bp upstream from the transcription start site [28, 29].

**Figure 3:**
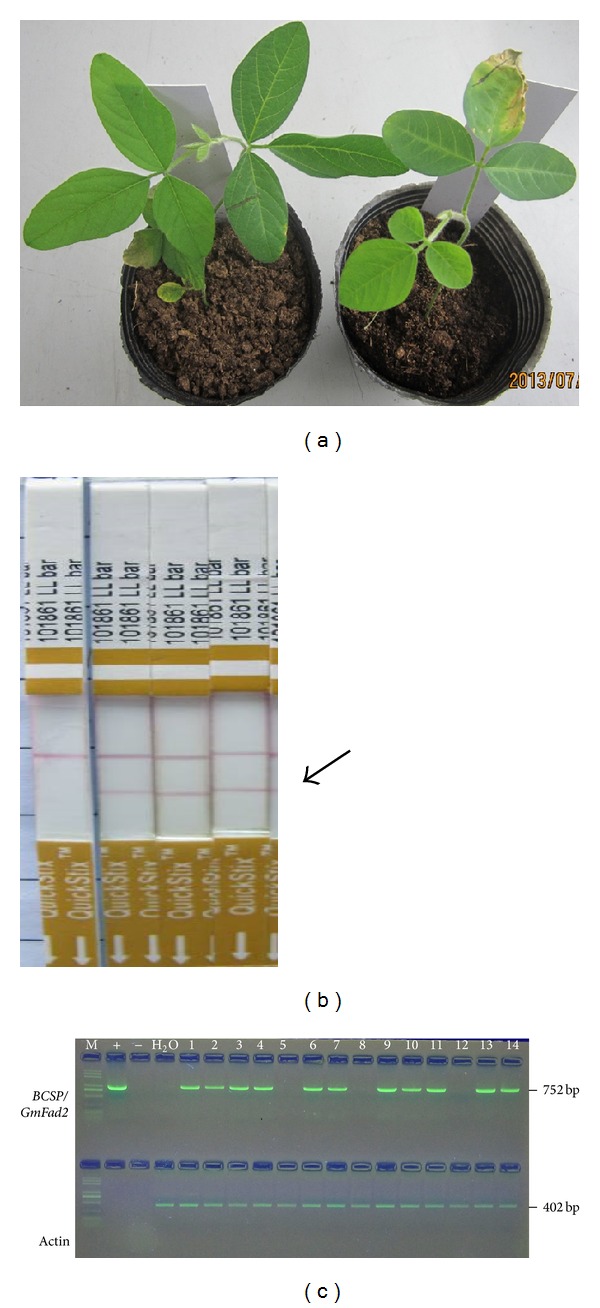
Procedure for confirmation of transgenic plants. (a) Identification of the herbicide resistance of the leaves of transgenic soybean plants using 135 mg/L Basta. Left: negative transgenic plant; right: positive transgenic plants. (b) Events of the PCR positive lines were randomly selected and tested using LibertyLink strips. (c) PCR analysis of genome DNA of putative transgenic soybean plant. The length of PCR production was 752 bp. M: trans2K plus DNA marker; +: plasmid DNA; −: nontransformed soybean; H_2_O. 1–14: the transgene line.

For the detection of the transgenic soybean plants, we used three methods (the leaf painting, the LibertyLink strip analysis, and the PCR) to confirm the positive transgene plants. We performed 200 Williams 82 on the seeds, which contains about 327 explants infected by the cotyledonary-node method. Finally, we obtained 105 T_0_ transgenic regeneration plants and a total of 55 plants tested positive, giving a 5.5% transformation efficiency.  Figure 3 shows a part of detection results by leaf painting, LibertyLink strip, and PCR analysis. Although the T_0_ transgenic plants were generated in the lab, while the T_1_, T_2_ were generated in the field, the rapid and exact method for determining positive transgenic status was pivotal. The PCR detection of a selected marker gene is a traditional and effective way of doing this, although false positive results are sometimes observed. Therefore, we checked a part of the sequence between the* BCSP* and the anti-*GmFad2-1b* at about 752 bp in the vector for the identification of any transgenic plants (Figure 3(c)). However, the primary PCR testing is cumbersome and costly. Instead of the PCR procedures, a leaf painting method was chosen. As leaf painting directly detected the effect of a selected marker gene, it showed fewer false positive results than the PCR procedures. Furthermore, the leaf painting method was low in cost and easy to carry out (Figure 3(a)). Since the* GmFad2-1b* gene fragment did not code for a functional protein, no novel protein was produced from the* GmFad2-1b* gene cassette. However, the expressing cassette contains the* bar* marker gene, which is characterized by a resistance to the herbicide bialaphos. The LibertyLink strips could therefore be used to quickly determinate genetically modified plants containing the* bar* gene (Figure 3(b)). By the methods stated above, we identified 55 positive T_0_ transgenic plants from the total 105 T_0_ transgenic lines. We also used these detection methods to examine the T_1_ and T_2_ progenies of every independently transformed T_0_ line. 11 of the 55 positive T_0_ lines were further regenerated to T_1_ lines and then T_2_ lines. All 11 plants which exhibited Basta resistance also showed amplification of the* bar* gene in the PCR analysis (results not shown), thereby confirming the presence and expression of the transgene in transgenic the soybean plants.

**Figure 4:**
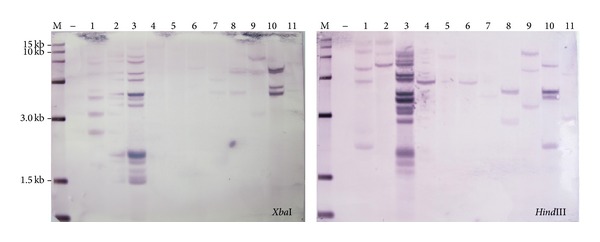
Southern blot hybridization of transgenic plants. The soybean genomic DNA was sampled from 11 independent events in the T_2_ generation (1–11) and digested with two different restriction enzymes,* Xba*I and* Hind*III, and hybridized with the bar probe labeled with DIG.

**Figure 5:**
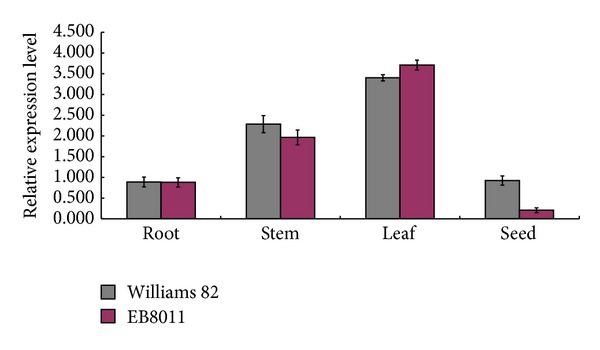
qRT-PCR analysis of* GmFad2-1b* in the different tissue. RNA was extracted from root, steam, leaf, and early flowering seed. The abundance of* GmFad2-1b* transcripts was normalized to that of* Actin* transcripts.

Consequently, because the* GmFad2-1b* gene and seed-specific promoter* BCSP* were endogenous genes, the marker gene* bar* in the pCAMBIA3300 vector became the probe for the Southern blot among the 11 T_2_ transgene lines. In addition, in order to guarantee the accuracy of the Southern blot, we used two different restriction enzymes,* Xba*I and* Hind*III, for digesting genomic DNA. Based on the Southern blot analysis and sequence data, it was determined that the* bar* gene cassette and the* GmFad2-1b* gene cassette have been inserted into the soybean genome (Figure 4). In previous studies there have been suggestions that if a high transgene copy number were integrated into the T_0_ plants, the alternative DNA copies should be integrated at different loci with a low recombination frequency [30]. This result was corroborated in our study by the Southern blot analyses of different T_2_ plants lines, EB8003, EB8005, EB8006, and EB8065, which presented at least four* bar* copies. One concern related to these high transgene copy numbers that were integrated in the host genome is the possibility of superfluous tandem integration, which could result in possible gene silencing and low recombinant protein production levels [29]. The lines EB8019, EB8011, EB8057, and EB8067 showed one or two copies, and the lines EB8007 and EB8008 cannot be confirmed by the two restriction enzymes at same time. All of the transgenic plants regenerated grew normally and set flowers and pods.

Increasing the oleic acid content in soybean seed oil is one of the most effective and efficient ways to enhance the nutritional value and practical utilization of soybean oil.

The overall lipid content of 11 T_2_ transgenic lines of soybean grain remained unchanged compared to the conventional soybean grain. Using fatty acid analysis, we selected three high oleic acid modified materials in the T_2_ transgene lines. They are EB8011, EB8057, and EB8061. The EB8011 soybean grain contains increased levels of oleic acid (up to 51.71%) and lower levels of linoleic acid (27.32%), linolenic acid (8.47%), and to a lesser extent palmitic acid (8.72%). In addition, EB8057 oleic acid content is 38.23%, and EB8061 oleic acid content is 40.48%, which were significantly higher than the receptor controls (17.70%) (Table 3). The lines EB8011 and EB8057 were presented at two copies in soybean genomic DNA while the line EB8061 was presented at three. In previous studies,* FAD2* genes were identified as encoding *ω*-6 fatty acid desaturases which catalyze the conversion of 18:1 oleic acid to 18:2 linoleic acid in soybeans [6, 11]. This conversion is of particular interest in the soybeans due to the growing desire for low linoleic soybean oil [4]. Moreover, a high oleic acid content is desirable because this monounsaturated fatty acid not only improves shelf life but also reduces the need for hydrogenation, a process which adds to the cost of the oil and generates unwanted trans fat that has been linked to many health problems in humans [1]. Our study demonstrated high-level suppression of* GmFad2-1b* by anti-RNA while also resulting in a high oleic acid in the soybeans.

## 4. Conclusions

We were successful in the suppression expression* GmFad2-1b* in transgenic soybean seeds. Based on the published results on soybeans, the soybean* GmFAD2* gene family has been previously characterized at the genome level for structure and expression [9–11] and also in genetic modification [15–19]. In our study, silencing of the endogenous* Fad2*-1 gene in seeds of the soybean event EB8011 was achieved through the introduction of a* GmFad2-1b* gene fragment driven by a seed-specific promoter. The transcription of the* GmFad2-1b* gene fragment in the seed acts to silence transcription of the endogenous* FAD2*-1 gene. The line EB8011 had a result of 51.17% oleic acid and 8.72% palmitic acid, in contrast to 17.7% oleic acid and 4.47% palmitic acid in the seeds. In future studies, we also expect that the altered ratio of oleic and linoleic acid will confer desirable properties on the resulting biodiesel, without a significant change in the yield.

## Figures and Tables

**Table 1:** Primers for different experiments.

Primer name	Primer sequence	PCR producer (bp)
GmFAD2-1b-F	5′-AGCCACTAGGCATGGGTCTAGCAAA-3′	801
GmFAD2-1b-R	5′-GCAATGGCACCCCATAAACACATAG-3′	
EB-R	5′-GGAAAGCAACCATATCAGCATATCAC-3′	752
EB-F	5′-TTCTCCAAGGTTGCATTCTTACTGG-3′	
GmActin-R	5′-TTGACTGAGCGTGGTTATTCC-3′	402
GmActin-F	5′-GATCTTCATGCTGCTGGGTG-3′	
qRTGmFad2-R	5′-CACCATTCACTGTTGGCCAA-3′	163
qRTGmFad2-F	5′-ATGAGGGAAAAGGGGTGAGG-3′	

**Table 2:** Transformation efficiency of soybean cotyledonary-node explants inoculated with *Agrobacterium tumefaciens*'s strain EHA101 harboring EB gene (pCamibia3300-*BCSP*-anti-*GmFad2*).

Number of experiments	Genotype	Explants infected	Plants surviving on SE medium	Positive T_0_ transformation	Transformation efficiency (%)
1	Williams 82	96	24	16	16.73
2	Williams 82	114	36	18	15.84
3	Williams 82	117	45	21	17.96

Total		327	105	55	16.84

**Table 3:** GC-MS results yielding the oil composition of the transgenic soybean seed line.

Sample	Generation	% palmitic	% stearic	% oleic	% linoleic	% linolenic
CK	Williams 82	10.49	4.47	17.70 ± 0.20	57.84	8.50
1	EB8003	10.63	3.58	14.96 ± 0.01	62.44	8.38
2	EB8005	10.50	4.02	16.22 ± 0.12	59.85	9.41
3	EB8006	10.59	4.20	16.85 ± 0.04	58.82	9.53
4	EB8007	10.33	3.95	17.07 ± 0.12	59.23	9.42
5	EB8008	10.53	3.80	15.39 ± 0.05	60.19	10.10
6	EB8019	11.13	4.10	16.55 ± 1.14	58.25	9.96
7	EB8011	8.72	2.93	51.17 ± 0.25**	27.32	8.47
8	EB8057	9.80	3.51	38.23 ± 0.05**	38.35	10.11
9	EB8061	9.19	3.76	40.48 ± 0.08**	37.44	9.12
10	EB8065	10.55	3.96	14.25 ± 0.09	60.63	10.61
11	EB8067	10.20	3.67	21.93 ± 0.05	54.03	10.17
